# The Double Burden of Obesity and Underweight in Yemeni Adults

**DOI:** 10.7759/cureus.50829

**Published:** 2023-12-20

**Authors:** Amran Ibrahim Mssode Ibrahim, Hind Bourkhime, Soumaya Benmaamar, Ibtissam El Harch, Nada Otmani, Sawsan Mohammed, Bouchra Benazzouz, Karima El Rhazi

**Affiliations:** 1 Biology and Public Health, Ibn Tofail University, Kenitra, MAR; 2 Chemistry and Pollution, Marine Sciences and Biological Research Authority, Al-Hudaydah, YEM; 3 Laboratory of Epidemiology, Clinical Research and Community Health, Faculty of Medicine and Pharmacy, Sidi Mohamed Ben Abdellah University, Fez, MAR; 4 Epidemiology and Public Health, Sidi Mohammed Ben Abdellah University, Fez, MAR; 5 Hematology, Faculty of Medicine and Health Sciences, University of Aden, Aden, YEM

**Keywords:** yemen, sociodemographic factors, underweight, overweight, obesity

## Abstract

Introduction: Yemen has a unique low-income population with several sociopolitical challenges and the association between weight disorders and sociodemographic and lifestyle factors is not clearly understood.

Aim: The aim of this study is to estimate the prevalence of obesity, overweight and underweight among Yemeni adults, and to identify their associated factors.

Methods: A cross-sectional study was conducted from 11 January to 25 March 2020, including 561 subjects of Yemen's adult population aged 18 and above, from four Yemeni governorates, who answered a questionnaire including demographic, socio-economic, and physical activity items after getting their signed consent. Height and weight were measured, and body mass index (BMI) was computed. The association between obesity or overweight (BMI ≥ 25.0kg/m^2^) or underweight (BMI < 18.5 kg/m^2^) and the other variables was analyzed using multinomial logistic regression.

Results: A total of 561 subjects aged ≥ 18 years have participated in this survey. The overall prevalence of obesity and overweight was 10.3%, 95% CI [7.7%; 12.8%] and 20.3%, 95% CI [17%; 23.5%] respectively, while the one for underweight was 21.2%, 95% CI [17.8%; 24.5%]. The risk of overweight-obesity increased with age (OR=1.02, 95% CI (1.01-1.03)), living in urban areas (OR= 1.680, 95% CI (1.105-2.552)) and average-high socioeconomic status (SES) (OR= 1.729, 95% CI (1.156-2.587)) while the risk of underweight decreased with the age (OR= 0.981, 95% CI (0.964-0.998)).

Conclusion: These findings provide a special case of high prevalence of obesity, overweight, and underweight in Yemen. Therefore, implementing awareness and prevention programs is highly recommended there.

## Introduction

Nowadays, weight disorders are presenting serious public health issues that are spreading at an alarming rate all over the world [1], especially in developing countries where the underweight and obesity combine in a dual existing burden [2]. Overweight and obesity have major health consequences, including non-insulin-dependent diabetes mellitus (NIDDM), coronary heart disease (CHD), hypertension, gallbladder disease, psychosocial problems, and certain types of cancer. Mortality from CHD has been shown to increase in overweight individuals, even at body weights only 10% above the average. The prevalence of hypertension in overweight adults is 2.9 times higher than that for non-overweight adults [1].

Obesity is a main risk factor for the onset of several chronic diseases. It has been linked to some hormone-dependent cancers, such as breast, colorectal, oesophagal, kidney, gallbladder, uterine, pancreatic, and liver cancer^ ^[3-4], and it's also considered a disease by itself [5]. Many environmental factors have a role in the onset of obesity [6], including lifestyle, rising incomes, urbanizing populations, increasing urbanization and globalization of food markets and foods with high contents of saturated fats and sugars, poor diet, a lack of physical exercise [7], age trends [8], and the socio-economic factors [7]. Dietary changes and inactivity patterns in transitional societies are inflaming the obesity epidemic [7]. On the other hand, being underweight can cause various pathologies such as nutritional deficiencies, weakened immune system, chronic fatigue, chronic diseases and infertility [9]. These weight disorders prevalence has changed significantly in all countries throughout the world in the past, and it is anticipated to continue in the future [10]. According to recent estimates, the global age-standardized prevalence of obesity grew from 3.2% to 10.8% in men and from 6.4% to 14.9% in women between 1975 and 2014, while the age-standardized global prevalence of underweight decreased from 13.8% to 8.8% in men and from 14.6% to 9.7% in women [10].

On a regional scale, the highest obesity prevalence is observed for women in Central Asia, the Middle East, and North Africa (31.4%) and for men in high-income Western countries (27.2%), while South Asia had the highest prevalence of underweight in 2014 (23.4% in men and 24.0% in women) [10]. It was estimated by the World Health Organization (WHO), in 2009 [11], that the prevalence of obesity in Kuwait tended into 36% in males vs 48% in females, in Saudi Arabia 28% vs 44%, in the United Arab Emirates 25% vs 42%, in Bahrain 21% vs 38%, in Qatar 19% vs 32%, in Lebanon 15% vs 27%, and in Oman 8% vs 17%. A few studies reported the prevalence of underweight in Gulf countries. It was estimated that 19.2% of females suffered from being underweight in Saudi Arabia (2014) [12], 13% of university female students in the United Arab Emirates [13], and 28.7% of females vs 19.3% of males in a Bahrain university [14]. Yemen is considered one of the poorest Arab nations and is experiencing a real humanitarian crisis, especially since March 2015, when it was plunged into a violent civil war [15]. This conflict has exposed its population to severe food insecurity, malnutrition, and even significant psychological stress, considered factors associated with being underweight [15-17]. In addition, since 1995, Yemen's societal progress has been accompanied by cultural shifts, increasing life expectancy, changes in nutritional habits and habitual physical activity, and other socio-economic factors. It was also associated with the reduction of communicable diseases, the increasing rate of non-communicable diseases (chronic diseases, most notably cancers, and CHD) and its associated risk factors such as hypertension, obesity, diabetes, and smoking [18,19].

A fact that makes this particular population face a double burden of weight disorders: obesity and underweight. In Yemen, few previous studies have investigated the prevalence of obesity and underweight in specific groups such as children, adults, and university students in an urban community [20-22]. A study conducted in 2012 estimated that the prevalence of underweight among Yemeni adults in an urban community was 16.6%, while overweight was 23.5% and obese was 8.8% [17]. Considering that it is extremely important to determine the prevalence and related correlates of obesity and underweight in Yemen's population, which could contribute to improving public health interventions and awareness programs, the aim of the current study is to estimate the prevalence of underweight and obesity in a nationally representative sample of Yemeni adults and describe its association with the socioeconomic status (SES) and lifestyle of subjects.

## Materials and methods

Study design and recruitment of participants

A cross-sectional survey was carried out from 11 January to 25 March 2020, on a national random sample of Yemen's adult population aged 18 and above, using a stratified two-stage sampling procedure. Four among 21 provinces representing the total urban and rural Yemeni territory were chosen at random to be the basis of this study, and neighbourhoods in both urban and rural areas were designated as a cluster. The total cluster choice was done proportionally to the distribution of the Yemen population, estimated in 2010, with around 50% of females, and more than 70% in the rural areas [23]. The participants aged 18 or older were randomly chosen from each cluster's household.

Ethical approval

The current study applied ethical recommendations based on the current rules for epidemiologic studies, which are compliant with the Helsinki Declaration [24]. It was approved by the Research and Ethics Committee, Faculty of Medicine and Health Sciences, University of Aden, Yemen. All subjects gave their consent before answering the survey.

Data collection

They were questioned through a comprehensive questionnaire consisting fundamentally of close-ended questions. To guarantee that the field survey was administered consistently and properly, survey administration training was provided to all field survey interviewers. Prior to embarking on data collection, a pilot study of the first 30 respondents was conducted to assess the validity, and reliability of the Arabic version of the questionnaire, to check also the clarity and ease of the questionnaire, and to verify data collection methods, particularly to determine the appropriateness of its format, level of difficulty, and length of enough time to complete the questionnaire. The questionnaire took almost 45 to 55 minutes to complete. The questionnaire consisted of the following items: socio-demographic variables, anthropometric measurements, and physical activity which was assessed through a detailed questionnaire by the Global Physical Activity Questionnaire (GPAQ) as recommended by WHO [25]. Variables studied sociodemographic and lifestyle variables. Occupations were categorized into two groups: active and inactive (retired, unemployed, and housewife). Marital status was grouped into two categories: married, unmarried (single, widowed or divorced). In addition, we grouped levels of education into the following categories: Illiterate, Koranic school and primary school, secondary and more (middle school, high school, and university). The SES was classified by self-report into two groups: low SES and average-high SES. Finally, physical activity was defined according to WHO criteria, which recommended that at least total physical activity MET minutes per week is >= 3000 for intense physical activity level, and at least five days of walking, and moderate or intense physical activity up to a minimum of 600 MET-minutes per week for moderate physical activity level. Low physical activity is considered if the value doesn't reach the criteria for either high or moderate levels [25]. Anthropometric measurements were taken three times for each participant and the average was calculated, we measured the body weight and the height. Bodyweight was measured using digital scales, with the individual dressed in light clothing and not wearing shoes. The weight of the body was measured in kilograms. The digital scales were calibrated before and during the study. Height was measured using a height gauge with the subject standing barefoot. Height was expressed in meters. Then, the body mass index (BMI) was calculated as recommended by WHO as body weight (kg)/square height (m^2^). According to WHO guidelines, BMI was classified as underweight (BMI < 18.5 kg/m^2^), normal weight (BMI 18.5-24.9 kg/m^2^), overweight (BMI 25-29.9 kg/m^2^) and obesity (BMI ≥ 30 kg/m^2^) [26].

Statistical analysis

A descriptive analysis was first performed and reported by the percentages for the qualitative variables, and the mean and standard deviation for the quantitative ones. In univariate analysis, the classical parametric tests (Chi^2^ test and Student test) were used to study the association between the different variables, and each category of BMI (obesity, overweight, normal and underweight). A multiple multinomial logistic regression was used to assess the determinant factors of underweight and obesity/overweight (normal weight as reference), taking into consideration confounding factors. The threshold for including variables in the model was set at a 20% level of significance. Associations were presented by an OR and its 95% confidence interval. A p-value < 5% is considered significant. All analyses were performed by the Statistical Package for the Social Sciences (IBM SPSS Statistics for Windows, IBM Corp., Version 20.0, Armonk, NY)

## Results

Basic characteristics

Table 1 shows the sample description of the main demographic and socio-economic characteristics. A total of 561 subjects aged ≥ 18 years were participated in this survey. Their mean age was 35.27 years ± 14.98. About 51.5% of participants were men, 70.4% lived in rural areas, and 85.6% were white-skinned. In general, in the overall Yemeni population, the prevalence of obesity, overweight and underweight was 10.3%, 20.3%, and 21.2% respectively (Table 1). In urban areas, the prevalence of obesity and overweight was significantly higher (18.7% and 25.3%, respectively) than in rural areas (6.8% and 18.7%, respectively, p =0.0001), while the prevalence of underweight was higher in rural areas (25.1% vs 12,0%). However, there was no significant association between gender and the different BMI classes (p= 0.982). The prevalence of obesity and overweight increased with age (p = 0.0001). Obesity and overweight were more common among married people vs unmarried (13.5%, 24.2% vs 3.4%, 11.9% respectively, p =0.0001), and the underweight was more common among unmarried people (29% in unmarried people vs 17.7% in married people, p= 0.0001). In addition, the prevalence of obesity, overweight increased with the SES (13.7%, 25.9% in average-high SES vs 8.3%, 16.9% in low SES respectively), while the underweight prevalence was higher in low SES compared to average-high SES (24.1% vs 16,5% respectively, p= 0.003) (Table 1). Finally, according to physical activity, the prevalence of obesity and overweight was lower among subjects who had high activity compared to those with low-moderate activity (8.7%, 17.1% vs 13.0%, 25.6% respectively, p= 0.01) (Table 1). Similarly, the prevalence of underweight was lower among subjects who had low-moderate activity compared to those with vigorous activity (16.7% vs 24.0% respectively, p= 0.010) (Table 1). However, the educational level (p= 0.858) and the occupation (p= 0.277) were not statistically associated with the BMI classes.

**Table 1:** BMI distribution by lifestyle and sociodemographic characteristics (N=561)

			BMI (kg/m^2^)			
		Total	Underweight <18.5	Normal weight 18.5-24.9	Overweight 25.0-29.9	Obesity ≥30.0
		N=561 (100%)	n=119 (21.21%)	n=270 (48.12%)	n=114 (20.32%)	n=58 (10.33%)
Age (years) N (Mean ± SD)		561 (35.27±14.98)	119 (30.78±14.69)	270 (34.39±15.1)	114 (39.72±13.92)	58 (39.83±13.85)
	p	0.0001				
Residency	Urban	166 (29.6%)	20 (12.0%)	73 (44.0%)	42 (25.3%)	31 (18.7%)
	Rural	395 (70.4%)	99 (25.1%)	197 (49.9%)	72 (18.2%)	27 (6.8%)
	p	0.0001				
Gender	Men	289 (51.5%)	63 (21.8%)	139 (48.1%)	58 (20.1%)	29 (10.0%)
	Women	272 (48.5%)	56 (20.6%)	131 (48.2%)	56 (20.6%)	29 (10.7%)
	p	0.982				
Skin Color	White	480 (85.6%)	100 (20.8%)	229 (47.7%)	98 (20.4%)	53 (11.0%)
	Brown	81 (14.4%)	19 (23.5%)	41 (50.6%)	16 (19.8%)	5 (6.2%)
	p	0.583				
Occupation	Active	233 (41.5%)	49 (21.0%)	108 (46.4%)	45 (19.3%)	31 (13.3%)
	Inactive	328 (58.5%)	70 (21.3%)	162 (49.4%)	69 (21.0%)	27 (8.2%)
	p	0.277				
Marital Status	Unmarried	176 (31.4%)	51 (29.0%)	98 (55.7%)	21 (11.9%)	6 (3.4%)
	Married	385 (68.6%)	68 (17.7%)	172 (44.7%)	93 (24.2%)	52 (13.5%)
	p	0.0001				
Educational Level	Illiteracy	213 (38.0%)	46 (21.6%)	108 (50.7%)	39 (18.3%)	20 (9.4%)
	Koranic-Primary school	123 (21.9%)	25 (20.3%)	54 (43.9%)	30 (24.4%)	14 (11.4%)
	Secondary and more	225 (40.1%)	48 (21.3%)	108 (48.0%)	45 (20.0%)	24 (10.7%)
	p	0.858				
Socioeconomic Status	Low	349 (62.2%)	84 (24.1%)	177 (50.7%)	59 (16.9%)	29 (8.3%)
	Average-High	212 (37.8%)	35 (16.5%)	93 (43.9%)	55 (25.9%)	29 (13.7%)
	p	0.003				
Physical Activity Level	Low-Moderate	215 (38.32%)	36 (16.7%)	96 (44.7%)	55 (25.6%)	28 (13.0%)
	High	346 (61.68%)	83 (24.0%)	174 (50.3%)	59 (17.1%)	30 (8.7%)
	p	0. 010				

After consideration of confounding factors (Table 2), the risk of overweight-obesity increased with age (OR=1.02, 95% CI (1.01-1.03)), living in urban areas (OR= 1.680, 95% CI (1.105-2.552)) and average-high SES (OR= 1.729, 95% CI (1.156-2.587)) while the risk of underweight decreased with the age (OR= 0.981, 95% CI (0.964-0.998)).

**Table 2:** Adjusted OR for underweight and overweight-obesity in a sample of adult Yemeni population, 2020 (N 561) Model adjusted for age, residency, marital status, socioeconomic status (SES), and physical activity.

BMI (kg/m^2^)		Underweight			Overweight-Obesity		
		OR	95% CI (OR)	p	OR	95% CI (OR)	p
Age (years)		0.981	[0.964; 0.998]	0.031	1.021	[1.008; 1.034]	0.001
Residency	Urban	0.598	[0.342; 1.046]	0.071	1.680	[1.105; 2.552]	0.015
	Rural	1	-	-	1	-	-
SES	Average-High	0.833	[0.519; 1.339]	0.451	1.729	[1.156; 2.587]	0.008
	Low	1	-	-	1	-	

## Discussion

In the present study, we assessed the prevalence of obesity, overweight, and underweight, in addition to related risk factors in a sample of the Yemeni population aged 18 years and older. The overall prevalence of obesity and overweight was 10.3% and 20.3% respectively, while the one for underweight was 21.2%. Overweight obesity was particularly associated with older age, living in urban areas, and average-high SES, whereas underweight was specifically associated with younger age. Otherwise, neither gender, colour, occupation, nor education level were associated with overweight obesity and underweight. The small sample size in our study may explain this. It is possible that Yemen's difficult situation has hindered the prioritisation of a healthy nutritional lifestyle. The prevalence of obesity and overweight in adults aged 18 years and older in the Yemeni population was lower than that estimated by the WHO in the worldwide population in 2016, which was 13% and 39%, respectively [7] and also lower than the WHO estimation for Yemen in 2008, which was approximately 17% and 46% respectively [16]. On the other hand, the prevalence of underweight among this population was higher than that estimated by the WHO worldwide population in 2016, which was approximately 9%, and also higher than the WHO estimation for Yemen in 2016, which was 7.7% [27].

Given these data, there has been a decrease in the prevalence of obesity and overweight since 2008 and an increase in the prevalence of underweight in the Yemeni population [17]. This may be attributed to poverty, malnutrition, low income, and high unemployment rates. The situation worsened after the war, which had a negative impact on the residents. Indeed, Yemen is one of the most impoverished Arab nations and has been characterized by a high level of malnutrition since March 2015, when the country was plunged into a violent civil war, a fact that exposed the population to suffer more from the underweight [6-15]. However, even if the prevalence of obesity and overweight decreased during the last decade, it remains potentially high and joins a global health crisis that may even be underestimated. This may be due to the fact that, as in other developing countries, Yemenis have more access to inexpensive foods that contain massive calories without nutritional value [28]. Regarding age, it was strongly associated with overweight obesity, which can be explained by many physiological mechanisms that may increase abdominal white adipose tissue and fat deposition in skeletal muscles [29], but more importantly by the fact that Yemeni elderly are more vulnerable to depression, disability, and lack of mobility due to the conflict, which may put them at greater risk of weight problems such as overweight and obesity [29,30]. Besides, Yemeni youth, most of whom are illiterate or without formal education, tend to work in informal sectors that recommend generally higher levels of physical activity [31]. This phenomenon, in addition to food insecurity and malnutrition [15-17], may explain why Yemeni youth are more likely to be underweight. In the major data available worldwide, the findings suggest that the prevalence of obesity is higher in urban areas [32], even in countries experiencing difficult political and socioeconomic conditions such as the population of the Palestinian West Bank (49.1% and 30.6% in urban women and men versus 36.8% and 18.1% in rural women and men, respectively) [33].

The results of this study provide the same information for the Yemeni population. Additionally, there was an increase in the urban prevalence of overweight and obesity from 2012 to the present (23.5% and 8.8% vs. 25.3% and 18.7%, respectively) [17]. This may reflect the emergence of new modern lifestyles in developing countries, involving a decrease in physical activity and overconsumption of inexpensive, calorie-dense foods [17]. Various research results support that obesity and overweight are more prevalent in the population living with higher SES in low-income countries [34]. In fact, this investigation supplies these outcomes and this may be a result of their access to comfortable lifestyle choices and more abundant food resources [34]. Therefore, a more affluent population may consume large portions of foods, especially carbohydrates and sugars, because they have the means to pay for them [34]. This is still the case, despite the conditions of war and famine experienced by the population since 2015 [16]. Also, this survey showed a significant difference in the prevalence of obesity according to marital status with more obesity in married people than in single ones. According to the rich literature, this could be due to various social factors and changes in eating behaviours [35]. Married people tend to be less active and prefer to eat together, thus increasing food supply and consuming more calories than single people [36]. This may also be related to the fact that single people tend to engage in unhealthy behaviours and have poorer nutrition outcomes compared to married people whose family structure encourages regular nutrition programs [37,38]. Several studies have highlighted the importance of the relationship between obesity and physical activity [39]. There is evidence that physical activity increases people's total energy expenditure and helps them stay in energy balance or even lose weight, as long as they do not eat more to compensate for the extra calories they burn [40]. This study provides the same direction of this association. A recent systematic review published in 2014 has studied the relationship between education level and obesity. This association wasn’t enough evident and it changed with the gender and country’s SES [41], in contradiction with many studies which show that highly educated people tend to have healthier lifestyles and be less obese [42,43], while the existing literature does not really provide sufficient data on the relationship between underweight and education level. Perhaps the small sample size of the learners in our study can explain this. It is possible that, due to the difficult situation that the Republic of Yemen has faced, they did not prioritize following a healthy nutritional lifestyle [44]. Likewise, despite the fact that the prevalence of obesity and overweight differ between women and men in low-income countries, as known from the literature [34], this association with gender was not evident in our context. This can perhaps be explained by the other problems that have pervaded Yemen in recent years, especially malnutrition and food insecurity, which make both sexes equally vulnerable [16].

Limitations

This study has a few limitations that should be mentioned. First, Yemen's socio-political conditions have been severely impacted by the ongoing war, resulting in numerous obstacles. Secondly, due to the descriptive character of the study, this could not give a real profile for *The Double Burden of Obesity and Underweight in Yemeni Adults*. Third, we did not have the chance to engage enough participants as reported in similar previous literature, which was a real barrier to demonstrating a significant association with some variables such as gender. Nevertheless, a heterogeneous sample was selected from four cities in Yemen which would lead to the best representation of the Yemeni population. The results could therefore be extrapolated to the whole of Yemen. Furthermore, the highlight of this national study was, to the best of our knowledge, the first survey of its kind on the prevalence of obesity and associated sociodemographic and lifestyle factors among adults in urban and rural areas of the Republic of Yemen including physical activity as a variable and could be of great added value for the national and international level.

## Conclusions

In conclusion, this community-based study shows a special case of high prevalence of obesity and overweight coupled to a high prevalence of underweight in the general population of Yemen. Getting older and living in an urban area with higher SES are the main sociodemographic and lifestyle factors that explain obesity. Conversely, young age correlated especially with underweight. Others studies are needed to confirm these results in particular in the context of the vulnerability such as that for Yemen. The recognition of these factors can facilitate planning of awareness programs and implementation of obesity reduction methods in this society.
